# Engaging Patient Advocates to Reduce Breast Cancer Disparities: Opportunities to Advance Breast Cancer Research and Engagement

**DOI:** 10.1007/s13187-025-02645-8

**Published:** 2025-06-20

**Authors:** Megan C. Edmonds, Maimah Karmo, Belinda Paulicin, Lizzie Wittig, Karla Funez, Ayres Warren, Sue Steele, Amanda Espenshied-Reilly, Shanda Cooper Goff, Virginia Leech

**Affiliations:** 1 Department of Social and Behavioral Science, Virginia Commonwealth University, Richmond, VA USA; 2 Icahn School of Medicine at Mount Sinai, New York, NY USA; 3 Tigerlily Foundation, Reston, USA; 4 Amherst College, Amherst, USA

**Keywords:** Black women, Patient-advocates, Breast cancer survivor, Survivorship, Health disparities

## Abstract

Inclusion of patient advocates in breast cancer (BC) research as experts, co-creators, and decision-makers has the potential to reduce breast cancer health disparities and enhance the implementation of clinical research. However, patient-advocates are often not included throughout the cancer care continuum. In this study, we examine the strategies of our patient advocacy training model to increase biomedical knowledge, address patient health literacy, empower patients, and lower access barriers, to facilitate participation in genomic research among Black women in five regions in the USA with the highest breast cancer disparities for Black women. This is a secondary analysis of patient advocates who were recruited to complete a breast cancer advocacy training program. Advocates were surveyed on the following topics: breast health knowledge, breast cancer screening, and diagnostic knowledge self-efficacy to advocate in medical settings. In this cross-sectional study design, we conducted a bivariate analysis using chi-square test to compare and describe participants’ pre- and post-survey responses after completion of the advocacy training. With frequencies and means, we summarized participants’ demographic factors, perceptions, and healthcare access barriers, such as participation in genetic counseling and testing. A total of 107 (86%) participants were Black women, with a mean age of 43 (8.5 sd). Breast cancer knowledge was 25% higher after completing the advocacy training compared to pre-training assessments (*p* < .001). Breast cancer screening diagnostic knowledge was 20% higher after completing the advocacy training compared to pre- training assessments (*p* < .001). Similarly, we saw a 24% increase in participants self-efficacy in medical settings (*p* < .001). This article provides an overview of training strategies involving patient-advocates’ role as needed experts within cancer disparities and cancer research. The advocacy training materials increased breast cancer screening knowledge and willingness to engage in community outreach activities to address breast cancer disparities. Findings from this work highlight the impact of educational training strategies to optimize patient-advocate participation and self-empowerment in cancer control and prevention with a focus on breast cancer related knowledge, mammography screening, and advocacy during medical encounters.

## Introduction

Breast cancer is the leading cause of cancer mortality for Black women compared to all racial/ethnic groups of women [1–3]. Reasons for breast cancer survival disparities are multifaceted across the continuum from diagnosis to treatment, involving underlying tumor biology, diagnosis at later stages, lack of healthcare access, and delays to systemic treatment in Black women compared to White women [4–6]. Reducing breast cancer disparities across this continuum is a public health priority for the National Cancer Institute and the White House Cancer Moonshot 2.0. Involving patient advocates in cancer research implementation and cancer prevention and control programming is an important strategy to help alleviate breast cancer disparities. Despite evidence that has shown advancements due to patient advocate engagement in the research process [7, 8], integrated partnerships with patient advocates are still lacking throughout the full lifecycle of the cancer continuum as advisors, trusted experts, and co-creators in research and science [7, 9, 10]. In this paper, we detail the development and implementation of a breast cancer-focused advocacy and empowerment training program for Black women. The training program goals were focused on increasing advocates’ biomedical knowledge, self-advocacy in medical encounters, and building community partnerships to advance health equity in breast cancer outcomes.

Collaborating with patient advocates in breast cancer research has the potential to reduce gaps in breast cancer disparities. Advocates are critically important because they share the same mission and offer opportunities to sustain research programs, nurture the bridge between patient-physician medical encounters, enhance educational tools, work to shift generational mistrust in healthcare, serve as peer leaders for empowered health, and establish a broader reach within communities. Prior research [7, 8, 11] has shown that patient advocates advance common and shared goals in research by enhancing outreach to diverse communities, serving as authentic and trusted voices from communities, and promoting engagement with stakeholders to accelerate personal, familial, community health and research goals [7, 12, 13]. One study reported that working with advocates helped to identify the current unmet needs in prostate cancer care delivery, which clarified the direction of future research needs [14]. Similarly, national organizations, such as the American Association for Cancer Research (AACR), report that the engagement of advocates with scientists helps to enhance the translation of clinical research into practice [8]. Additionally, other studies have reported research advances from advocacy involvement with regard to increased recruitment, and engagement in clinical cancer research, which helps translate into better study implementation outcomes [15–17]. However, when training advocates in breast cancer prevention and control, sustainable practices, engagement strategies, and efficacy outcomes are less understood.

In this paper, we present effective engagement strategies in the breast cancer prevention field through the lenses of a non-profit organization that works and trains patient advocates to provide robust breast cancer education, awareness, and advocacy to young women diagnosed with breast cancer. More specifically, our study fills an important research gap with regard to sustainable engagement strategies to enhance outcomes for a breast cancer focused advocacy training program. The goal of Tigerlily’s Advocate Now to Grow, Empower and Lead (ANGEL) LEAD pilot advocacy program was to address breast cancer disparities across five regional areas: Oakland, California; Washington D.C.; Pennsylvania (Philadelphia and Pittsburgh); New Orleans, Louisiana; and Southeast Michigan (focused on Flint and Detroit) [18, 19].

## Methods

### Study Setting

**Fig. 1:**
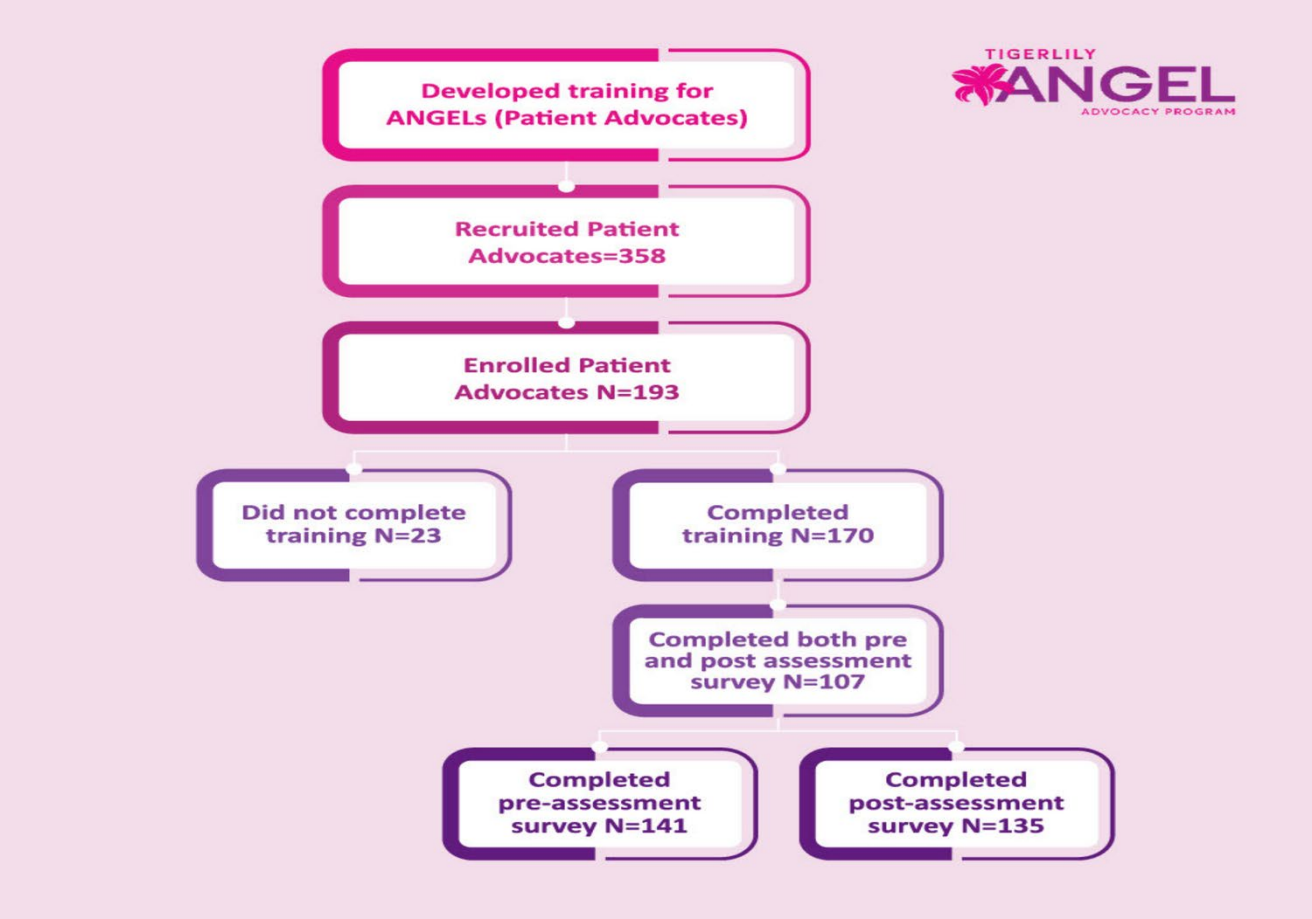
ANGEL study schema

The goal of this cross sectional study was to highlight the outcomes of a breast cancer-focused virtual advocacy training program. Tigerlily Foundation’s ANGEL LEAD pilot program is a patient advocate training program established in 2021, with a goal to help reduce breast cancer inequities within five regional counties with the highest breast cancer mortality for Black women [18, 19]. Participants for the ANGEL Advocacy program were recruited online into 17 cohorts across five regional areas. The recruitment process occurred virtually and in-person through several outreach efforts including an ANGEL Advocacy website, online social media outreach, community advocacy groups, and referrals. Advocates were eligible to participate in the program if they met the following criteria: personal history of breast cancer and/or caregiver or healthcare worker in the breast cancer field, between the ages of 21 and 50, self-identified as a woman, self-identified as a person of color, and residency within one of the five regional areas. The full details of our recruitment procedures can be found online on the website. Of 170 enrolled participants, 63% completed training assessments and were consented by program staff leaving a final analytical sample of 107 for this study (Fig. 1). Consented participants were incentivized with a welcome kit when they were enrolled into the program, and a graduation gift upon completion of the training and pre-and post-training survey instruments. Institutional Review Board (IRB) approval was obtained to conduct the study analysis. Figure 1 illustrates the study schema.

### Engagement Strategies and Training Implementation

**Fig. 2:**
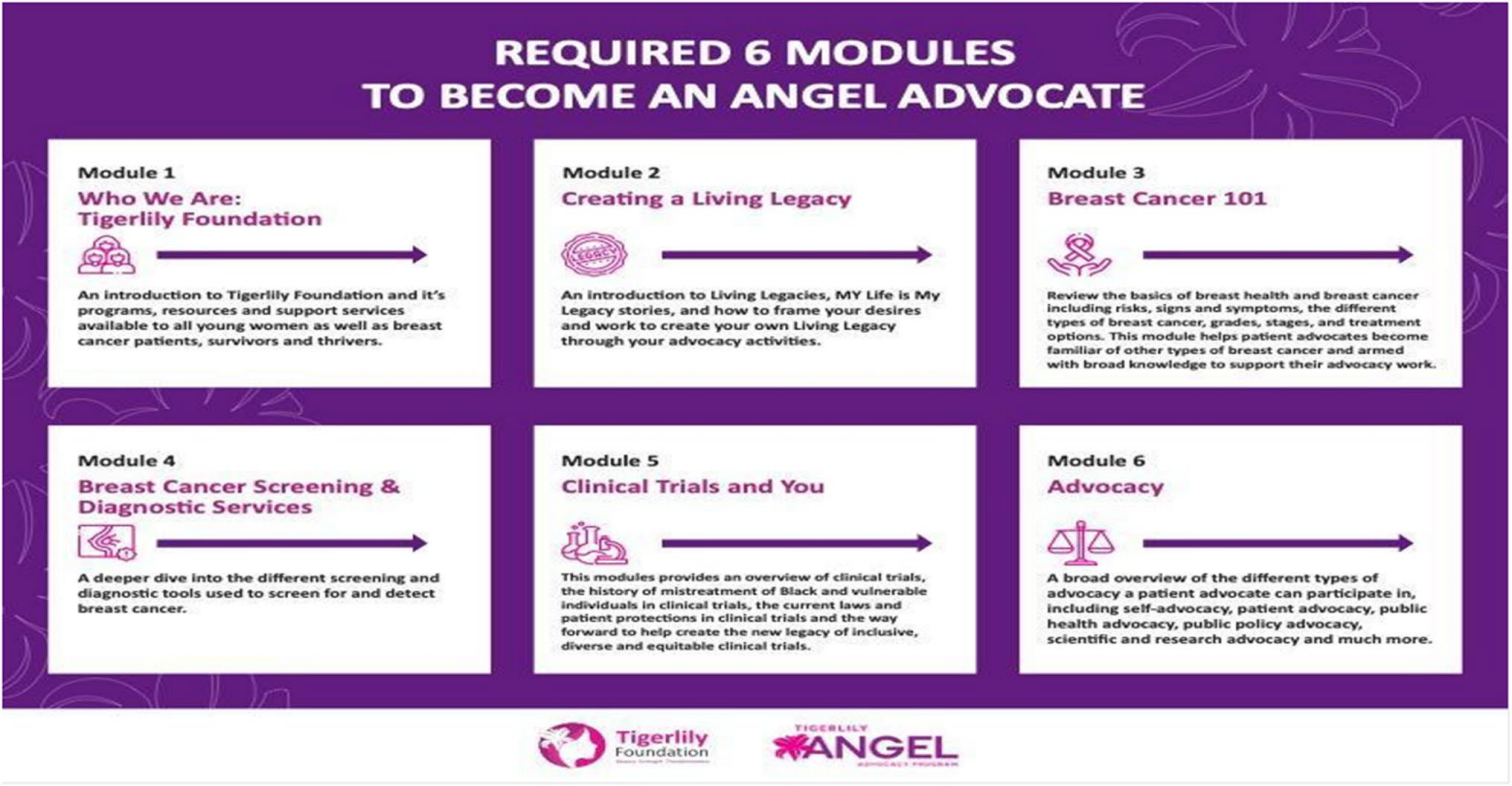
ANGEL Advocate Required Training Modules

Recruitment of advocates consisted of several engagement strategies with community partners, health care providers and snowballing methods (Table 1). After completing the online application for the ANGEL Advocacy program, eligible participants were invited into a cohort to complete six online training modules (Fig. 2). Advocates were given an online portal login to access the modules through a virtual learning management portal. A trainer led the modules virtually on a web-based platform on the following topics: breast cancer risks, signs and symptoms, breast cancer screening and diagnostic services, genetic testing, clinical trials, advocacy (including self-efficacy) and healthcare discrimination (Fig. 2). The modules were 1.5-h webinars accessible through the online portal (Fig. 2). Advocates in each cohort were informed about their responsibilities and expectations through a contract with the following guidelines: (1) attend live virtual trainings; (2) to not miss more than two trainings; (3) lead breast cancer educational engagement activities (e.g., speaking engagements, community events); (4) complete the baseline survey before beginning the training modules; and (5) complete the post-survey after each training module. To prioritize engagement and recruitment of advocates regionally, we established a peer-support ANGEL lead initiative in five regional areas with higher breast cancer mortality rates for Black women. The purpose of the ANGEL lead initiative was to recruit a trusted messenger within the local areas with the following expectations: maintain permanent residence in one of the five regional areas; self-identify as a Black woman with a personal history of breast cancer; self- advocate in their community, in policy, and involved in breast cancer research activities; educate their local community through speaking engagements; participate in health events; educate their virtual community through social media; and serve as thought leaders to Tigerlily’s partners. Study outcomes were defined as completion of training, and all survey measures (baseline, pre-and post-surveys). To evaluate advocates’ readiness and confidence in the training materials, participants were asked to complete pre-training and post-training assessments on the following topics: knowledge of biomedical information, their attitudes and beliefs toward being a patient advocate, healthcare access barriers, and their participation in breast cancer research and policy. To encourage advocates to complete pre and post training survey measures, each participant received personalized emails and text reminders as an engagement strategy, from their ANGEL lead.

**Table 1:** Characteristics of the sample *N* = 107

Variables	*N* (%)
Age (m ± sd)	43 ± 8.5
Race	
Black or African American	93 (86.9)
Other	14 (13.0)
Breast cancer stage	
Early stage (0–II)	91 (85)
Metastatic	16 (14.9)
BRCA 1 /2 genetic testing referral	
Yes	82 (76.4)
No	25 (23.3)
Healthcare barrier	
Health insurance	37 (35)
Patient-centered communication with medical providers	52 (48)
Clinical health information for women	61 (57)
Mammography screening navigation	47 (44)
Clinical trial knowledge	
Familiarly with the process of participating in a breast cancer clinical trial	16 (14.9)
Understand the value of participating in clinical trials for Black and Indigenous people of color	57 (53.2)
Knowledge to assess a clinical trial to participate in comfortably	26 (24.7)

#### Survey Measures

Advocates were asked to complete a baseline survey that included sociodemographic factors (age, race, breast cancer history). The pre- and post-training survey assessed participants’ breast cancer knowledge with the question “how would you rate your knowledge of breast cancer information (i.e., causes, symptoms, testing, treatments and resources)?” Responses were measured on a 4-point Likert scale ranging from “Below Average to Excellent,” where Excellent indicated greater breast cancer knowledge. Participants self-efficacy in feeling empowered to advocate for their health during medical encounters with physicians was measured with responses ranging from “Almost Always, Sometimes, Often or Never” likely to speak up in medical encounters. Participant’s receipt of genetic testing referral was measured with a yes/no question, “Has your oncologist or surgeon ever recommended BRCA 1/2 testing or related genetics or biomarker screening?” Participants were asked to indicate a yes/no response on their experience with health care barriers covering the following topics: health insurance, patient-centered communication with medical providers, clinical health information for Black women, and mammography screening navigation. Participants knowledge about breast cancer screening was measured with responses ranging from “Very familiar to Not at all,” where higher scores indicated more BC screening knowledge. Participant’s knowledge to access a clinical trial for participation was measured with a yes/no question. Clinical trial knowledge was measured with two questions, “Are you familiar with the process of participating in a breast cancer clinical trial?” and “Do you understand the value of participating in clinical trials for Black and Indigenous people of color?” where responses were yes/no.

#### Survey Analysis

Characteristics of the sample were described using descriptive statistics (frequencies and std). To describe the sample and factors associated with completing the patient advocate training, in bivariate analyses, chi-square tests (for categorical variables such as stage at diagnosis), and *t* tests (continuous variables, e.g., age) were conducted. *p*-values were based on two-sided tests and were considered significant at 0.05 or lower. All statistical analyses were conducted using SAS version 9.

## Results

Of 107 women, 93 (86%) were African American breast cancer survivors with a mean age of 43 (8.5 sd) (Table 1); 91 (85%) were diagnosed with earlier stage breast cancer (stage 0–II) with 16 (14%) diagnosed with metastatic disease; 82 (76%) of breast cancer survivors received BRCA 1/2 genetic testing. Advocates reported the following healthcare barriers before completing the ANGEL training program: health insurance 37 (35%), clinical health information for Black women 61 (57%), mammography screening navigating 47 (44%), and communicating with clinical providers 52 (48%).

In Table 2, we describe the outcomes from the ANGEL training program. As a result of participation in the training program, advocates readiness to communicate screening diagnostic risk information improved by 25% (*p* < 0.001). Breast cancer screening diagnostic knowledge was 20% higher after completing the advocacy training compared to pre-training assessments (*p* < 0.001). Similarly, we saw an increase post training in participants’ intent to self-advocate for self and others in medical settings with a 24% increase (*p* < 0.001).

**Table 2:** Pre- and post-assessments for ANGEL participants *N* = 107

Variables	Pre (*N*%)	Post (*N*%)	*p*-value
Breast cancer knowledge			< 0.0001
Excellent	5 (4.6)	26 (24.7)	
Above average	40 (37.3)	58 (55.2)	
Average	52 (48.6)	20 (19.0)	
Below average	10 (9.3)	1 (0.9)	
Breast cancer screening knowledge			< 0.0001
Very familiar	5 (4.6)	19 (18.1)	
Moderately familiar	28 (26.17)	68 (64.7)	
Not at all	74 (69.16)	18 (17.1)	
Self-efficacy to speak up in medical encounters			< 0.0001
Almost always	69 (65)	93 (89.4)	
Often	25 (23.8)	9 (8.6)	
Sometimes	11 (10.4)	2 (1.9)	

**Fig. 3:**
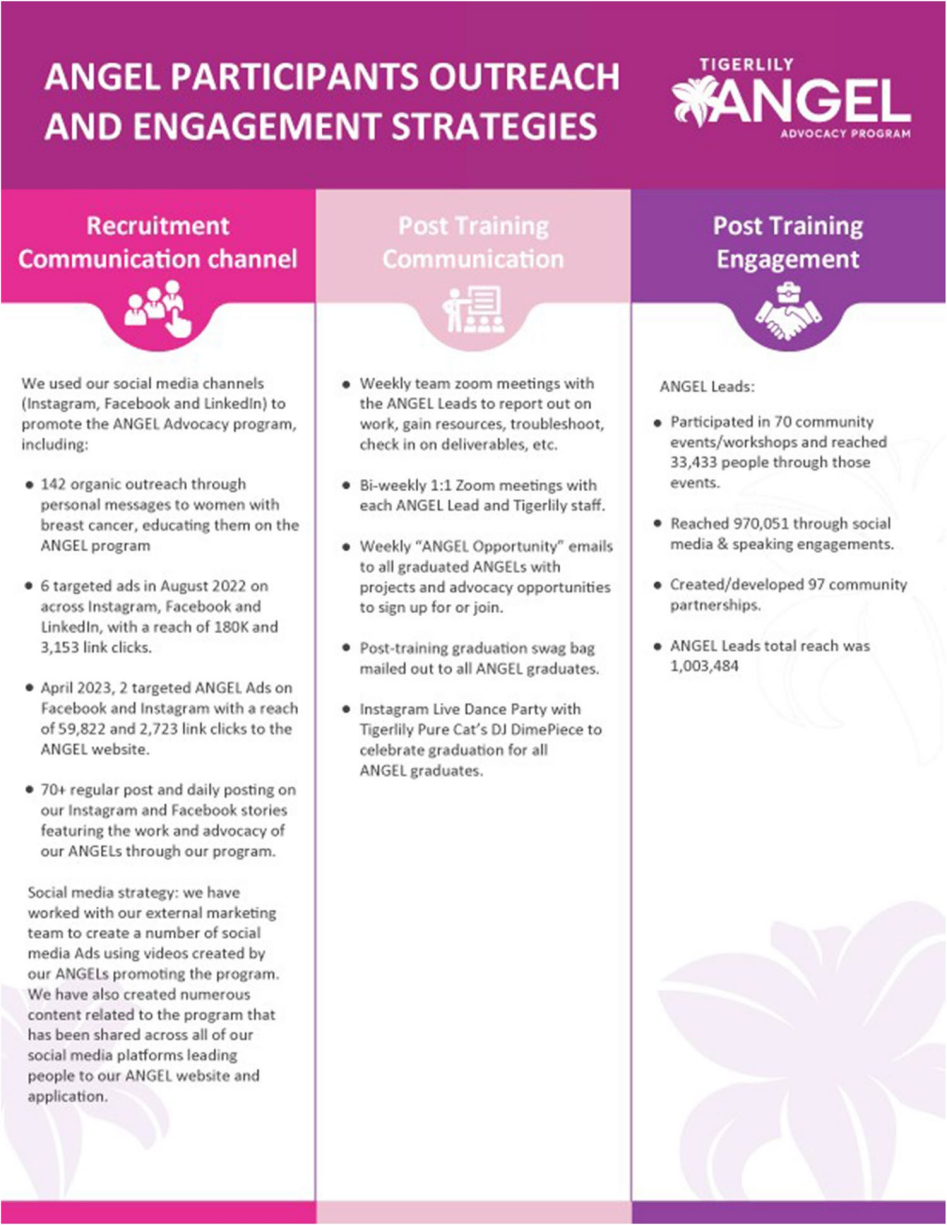
Outreach and engagement strategies

 78% of advocates were more likely to complete the training when using engagement strategies which included personal emails from ANGEL leads, bi-weekly zoom meetings, and connection with community health care partners and other patient advocates (Fig. 3). Of those who completed the training, an impressive 78% reported feeling empowered with self-advocacy skills and took initiative to lead community-level outreach activities for recruitment and education by hosting community events and distributing resources to women in their community. Engagement at the local level included distributing brochures, speaking engagements, and social media shares for their assigned ANGEL community members.

## Discussion

We developed a breast cancer advocacy and empowerment training program to effectively communicate breast cancer risk reduction strategies for Black women residing in five regions with the highest breast cancer mortality rates. The goal of the training program was to improve biomedical knowledge, address patient health literacy, and increase self-empowerment. Our training increased women’s knowledge in the following areas: breast cancer screening, diagnostics, and the willingness to advocate for themselves and others in medical settings. Women who completed our training led multiple community outreach activities throughout the five regions at the local, state, and national level to address health disparities and dismantle barriers to breast cancer care.

Integrating community advocates and medical advocacy is critically important for the advancement of clinical research in the cancer prevention and control field to help address cancer disparities and access barriers [20–22]. For the last 20 years, national organizations, such as the American Association for Cancer Research (AACR), have engaged advocates with scientists at annual meetings for continued education and training [8]. Similarly, Patient-Centered Outcomes Research Institute (PCORI) requires community advocate collaboration in their funding announcements, where researchers are required to identify and name qualified advocates as co-investigators on their project submissions [8]. With this same perspective, we developed our breast cancer advocacy training program in concert with community partners in health care to recruit potential advocates across five regions with the worst breast cancer mortality outcomes for Black women in our nation. Much of our success of reaching over 170 predominately Black women was attributed to our rapport with community and organizational partners, as well as our social media news blast presence. We recommend scientists and organizations looking to grow their connections with advocacy partners in biomedical research, to identify strategies that engage them with reputable liaisons and trusted community members in their region and to grow their social media presence to increase outreach and engagement.

When training advocates in breast cancer prevention and control, sustainable practices, engagement strategies, are necessary for efficacy outcomes. In our study, engagement strategy inclusion of ANGEL leads assisted with our accrual, implementation, and success of our training program. Similarly, a recent study found that advocates with a personal history of cancer had significantly greater participation in cancer control training workshops [15]. Another study found that after training advocates on hereditary breast cancer, their confidence and willingness to conduct educational sessions increased within community settings [23]. While these studies highlight advocate participation and demographic factors, optimal strategies to sustain these efforts after study implementation are needed to advance the translation of cancer research and to improve cancer control. Tigerlily’s work highlights a sustainable training model that optimize community partnerships that lead to patient-advocate participation and self-empowerment in cancer control and prevention, with a focus on breast cancer-related knowledge, mammography screening, and advocacy during medical encounters [7, 24, 25]. The Tigerlily Foundation has recruited, trained, and engaged more than 400 women in breast cancer prevention related programming. Specifically, Tigerlily has trained 358 women for the ANGEL advocacy program beyond the five regions. This outcome highlights the program’s potential to sustain effect positive change at the individual level, empowering women with the skills and confidence needed to navigate various aspects of their lives. We encourage more community partnerships in regions and rural communities to foster trust, and ensure biomedical research addresses the needs and priorities to mitigate cancer mortality in underserved communities.

Some strengths to note that optimized our reach and engagement for our training program was our strong social media presence and rapport with community partnerships, which served as our top two engagement strategies. Limitations that we could not control were loss of follow-up. Some women could not complete training or assessments due to progression of illness, schedule changes, or changes in life circumstance. Women who did not have access to computers had barriers to effectively participate in online trainings. The use of smart phones sometimes posed challenges for learning the materials. To sustain our engagement and work with our trained advocates on future initiatives, we maintain a weekly advocacy opportunity email with the ANGEL Advocates. We also continued training on extended topics and host regular opportunities to virtually gather as a community. This nurtures relationships with both non-clinical community stakeholders and clinical stakeholders as advocacy opportunities for the ANGELs. The program now has 97 non-clinical community stakeholders and more than 3,500 clinical stakeholders and partners, engaged in over 45 community events with a total reach of 30,000 + community members with 200,000 + reached through social media and speaking engagement activities, the ANGEL program has demonstrated its capacity to engage diverse groups in its mission.

## Conclusion

There is limited breast cancer risk reduction programming aimed to increase patient advocates to advance biomedical science and address breast cancer disparities. Findings from this study determined that engagement strategies of ANGEL leads within five regional areas with the highest breast cancer mortality helped to sustain advocacy programming, promote breast health knowledge, and improve our overall accrual, implementation, and success of the training program. Our advocacy training increased women’s breast cancer screening and diagnostic knowledge and their willingness to advocate for themselves and others in clinical settings. To increase inclusion of patient advocates in biomedical research and clinical practices, strategies may include targeting local, trusted messengers to deliver and sustain cancer prevention programming.
